# Limitations of three-dimensional power Doppler angiography in preoperative evaluation of ovarian tumors

**DOI:** 10.1186/s13048-015-0174-y

**Published:** 2015-07-29

**Authors:** Liliane Silvestre, Wellington P. Martins, Francisco J. Candido-dos-Reis

**Affiliations:** Department of Gynecology and Obstetrics, Ribeirao Preto School of Medicine, University of Sao Paulo, Av. Bandeirantes 3900, 8o andar, Ribeirao Preto, 14049-900 Brazil

**Keywords:** Ovarian neoplasms, Adnexal diseases, Ultrasonography, Doppler, Blood flow velocity

## Abstract

**Background:**

This study describes the accuracy of three-dimensional power Doppler (3D-PD) angiography as secondary method for differential diagnosis of ovarian tumors.

**Method:**

Seventy-five women scheduled for surgical removal of adnexal masses were assessed by transvaginal ultrasound. Ovarian tumors were classified by IOTA simple rules and two three-dimensional blocks were recorded. In a second step analyses, a 4 cm^3^ spherical sample was obtained from the highest vascularized solid area of each stored block. Vascularization index (VI), flow index (FI) and vascularization-flow index (VFI) were calculated. The repeatability was assessed by concordance correlation coefficient (CCC) and limits of agreement (LoA), and diagnostic accuracy by area under ROC curve.

**Results:**

IOTA simple rules classified 26 cases as benign, nine as inconclusive and 40 as malignant. There were eight false positive and no false negative. Among the masses classified as inconclusive or malignant by IOTA simple rules, the CCCs were 0.91 for VI, 0.70 for FI, and 0.86 for VFI. The areas under ROC curve were 0.82 for VI, 0.67 for FI and 0.81 for VFI.

**Conclusions:**

3D-PD angiography presented considerable intraobserver variability and low accuracy for identifying false positive results of IOTA simple rules.

## Background

Ovarian cancer is the leading cause of death among gynecological cancers [1]. Because the patient survival is strongly associated with disease stage, there have been many attempts of early diagnosis. While the occurrence of masses in adnexal region is very frequent (up to 20 %), the prevalence of ovarian cancer is low (approximately 0.2-0.3 % in post-menopausal women). To detect one ovarian cancer case using screening methods, approximately 5 women will be submitted to surgery [2, 3]. Tranvaginal ultrasonography is one of the most evaluated methods for early detection of ovarian cancer. In clinical practice, detected adnexal masses can be classified according to International Tumor Ovarian Analysis (IOTA) “simple rules”. IOTA simple rules are based on the identification of some simple features during conventional ultrasound examination and presents high sensitivity [4]. However, the false positive rate is elevated. About 40-50 % of the women with inconclusive and malignant results by IOTA simple rules have benign ovarian tumors [5, 6].

Three-dimensional ultrasonography allows quantifying blood flow and vascularization in ovarian tumors [7]. Vascularization index (VI), flow index (FI) and vascularization-flow index (VFI) can be calculated after manual outlining of vascularized solid areas within the tumor [8] or after spherical sampling from the most vascularized area of the tumor, the area is selected by the examiner and the spherical sampling performed automatically by the software [9]. The results are similar for both methods, however virtual spherical sampling saves time compared with manual sampling when the examiner is less experienced [10].

Differences in vascularization between malignant and benign ovarian tumors are well established [11] and vascular indices obtained using 3D-PD angiography are correlated with tissue perfusion as reported for experimental models [12–14]. However, the usefulness of vascular virtual biopsies for differential diagnosis of adnexal masses is still controversy [15, 16]. In this study, our objective was to evaluate whether 3D-PD angiography can be used to reduce the false positives among adnexal masses with inconclusive or malignant result according to IOTA simple rules.

## Methods

### Study design

We invited 75 women that were consecutively scheduled for surgery to remove adnexal masses from September 2008 to December 2010. All these women accepted to participate and were included in the study. The study was approved by the institutional review board (Ethics Committee of the University Hospital of Ribeirao Preto School of Medicine) and informed consents were obtained from all participants. Final diagnoses were based on the tumor histology, classified according to WHO criteria [17]. The manuscript was prepared following the STARD (diagnostic accuracy studies) guideline.

### Ultrasound

Participants were assessed by transvaginal ultrasound scan using a Voluson 730 (GE Healthcare, Zipf, Austria) ultrasound machine equipped with a volumetric vaginal probe (RIC5-9-D). The adnexal masses were classified as benign, malignant or inconclusive according to IOTA simple rules [18]. Briefly, if one or more malignant features were present in absence of benign feature, the mass was classified as malignant; if one or more benign features were present in absence of malignant feature, the mass was classified as benign; if both malignant features and benign features were present, or if no benign or malignant feature was present, the result was inconclusive. Vascular power Doppler score is included in IOTA simple rules as one variable: a score of 1 is given when no blood flow is found in the tumor, a score of 2 when only minimal flow is detected, a score of three when moderate flow is present and a score of four when the tumor presents marked blood flow [19].

After the conventional ultrasound, the same observer acquired two three-dimensional volumes from the adnexal masses. For large tumors, when it was not possible to completely include the tumor in the block, the sonographer chose the most complex area of the tumor to be sampled and attempted to sample the same area for both blocks. The following predetermined power Doppler settings that were kept constant throughout the study: (Main PD settings) Filter = low1; PRF = 0.6 KHz; gain = 0.0; Quality = normal; (Sub PD settings) Freq = mid; Flow res = mid1; Smooth = 5/6; Ensemble = 12; Line Den = 7; PD map = 5 (Gently Color = on); Balance = G > 200; Artifact = on; L. Filter = 2. The blocks were recorded for later analyses. The maximum interval time between ultrasound scan and surgery was 7 days; this was performed to reduce the risk of the occurrence/regression of a disease between the ultrasound scan and the surgery.

### 3D-PD angiography

A 4 cm^3^ spherical sample was obtained from the highest vascularized solid area of each tumor stored block using the 4D View software (version 5.0, GE Healthcare). The maximum distance between the ultrasound probe and the spherical sample was 4 cm (mean = 2 cm; average difference between distances from each pair of samples = 0.1 cm; effect size of intra-pair variation = 0.04). VI, FI and VFI were calculated. The observer was blinded for the histological result.

### Statistical analyses

Statistical analysis was conducted using STATA/SE version 13 (StataCorp). Mann–Whitney test was used to compare quantitative variables between benign and malignant groups, Fisher's exact test to analyze contingency tables and Kruskal-Wallis test to compare the 3D-PD indices among the tumors grouped by power Doppler scores. Intra-observer repeatability and diagnostic accuracy and were evaluated for 49 women with inconclusive or malignant results by IOTA. The intra-observer repeatability was assessed comparing the results of vascular virtual biopsies from the two blocks of each tumor by the concordance correlation coefficient (CCC) [20] and the limits of agreement (LoA) of the relative difference between measurements [21]. CCC values should be higher than 0.70 to be used as research tool, higher than 0.90 to be used in clinical practice, and higher than 0.95 for individual and important decisions [22]. The diagnostic accuracy of 3D-PD indices was estimated by the area under ROC curve.

## Results

The final histology of adnexal masses is presented in the Table 1, 32 (43 %) were malignant and 43 (57 %) benign. According to IOTA simple rules, 26 adnexal masses were classified as benign, 40 as malignant and 9 cases had inconclusive result. IOTA simple rules presented 100 % of sensitivity and 60 % of specificity. IOTA simple rules and 3D-PD angiography results are shown in the Table 2.

**Table 1:** Final diagnosis of 75 adnexal masses

Histology	N	%
Benign		
Brenner tumor	2	2.7
Dermoid cyst	8	10.7
Endometriotic cyst	11	14.7
Fibroma	4	5.3
Haemorragic cyst	3	4.0
Mucinous adenoma	8	10.7
Serous adenoma	6	8.0
Ovarian abscess sequelae	1	1.3
Malignant		
Clear cell carcinoma	1	1.3
Endometrioid carcinoma	2	2.7
Metastatic tumor	5	6.7
Mucinous bordeline tumor	2	2.7
Serous borderline tumor	3	4.0
Serous carcinoma	13	17.3
Stromal tumor	4	5.3
Transitional cell carcinoma	1	1.3
Undifferentiated carcinoma	1	1.3

**Table 2:** IOTA and 3D-PD indices according to final diagnosis

	Benign (N = 43)	Malignant (N = 32)	P
Age^a^	42 (18–82)	52 (20–78)	0.02
IOTA simple rules^b^			<0.0001
Benign	26	0	
Inconclusive	9	0	
Malignant	8	32	
VI^a^	4.5 (0.01–28.8)	19.6 (3.4–55.9)	<0.0001
FI^a^	38.1 (23.0–58.2)	47.3 (31.2–60.2)	0.0001
VFI^a^	1.7 (0.002–10.4)	9.1 (1.6–30.7)	<0.0001

^a^median (range), Mann–Whitney test

^b^Fisher's exact test

**Fig. 1:**
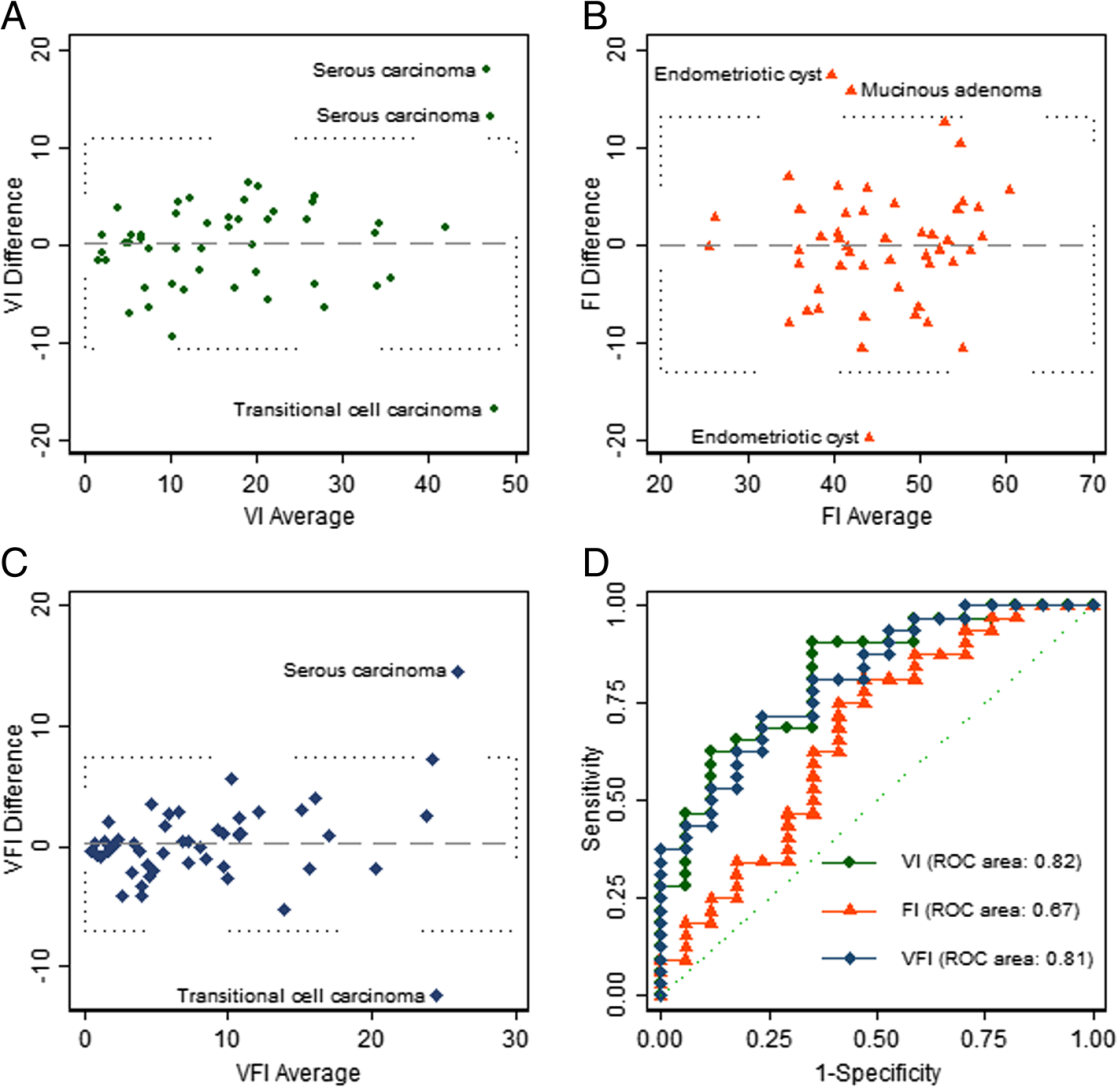
Repeteability and accuracy of 3D-PD indices in ovarian tumors with malignant or inconclusive IOTA results. **a** Bland and Altman plot for vascularization index (VI), **b** Bland and Altman plot for flow index (FI), **c** Bland and Altman plot for vascularization-flow index (VFI), **d** ROC curve for 3D-PD indices

Intra-observer repeatabilities of 3D-PD indices from virtual vascular biopsies in the group of 49 cases with malignant or inconclusive results according to IOTA classification are shown in Table 3. We would expect that 95 % of the differences between pairs of measurements would be less than 10.8 for VI, less than 13.0 for FI and less than 6.4 for VFI (Fig. 1).

**Table 3:** Repeatability analyses of the 3D-PD indices in the 49 adnexal masses with inconclusive or malignant results by IOTA simple rules: concordance correlation coefficient (CCC) and limits of agreement (LoA)

	CCC (95 % CI)	Mean difference (LoA)
VI	0.91 (0.84 to 0.95)	0.12 (−10.6 to +10.9)
FI	0.70 (0.53 to 0.82)	−0.02 (−13.1 to +13.0)
VFI	0.86 (0.77 to 0.92)	0.33 (−6.1 to + 6.8)

*VI* vascularization index, *FI* flow index, *VFI* vascularization-flow index

Diagnostic accuracies of 3D-PD indices for reclassifyadnexal masses with inconclusive or malignant results by IOTA simple rules are presented in Fig. 1d. We observed an area under ROC curves of 0.82 (95 % CI: 0.70-0.94) for VI, 0.67 (95 % CI: 0.50-0.84) for FI and 0.81 (95 % CI: 0.69-0.93) for VFI. The highest cut-offs still resulting in 100 % sensitivity for malignancy were: VI ≥ 3.4, FI ≥ 31.2 and VFI ≥ 1.6. The number of false positives was 14 for all 3D-PD indices.

**Fig. 2:**
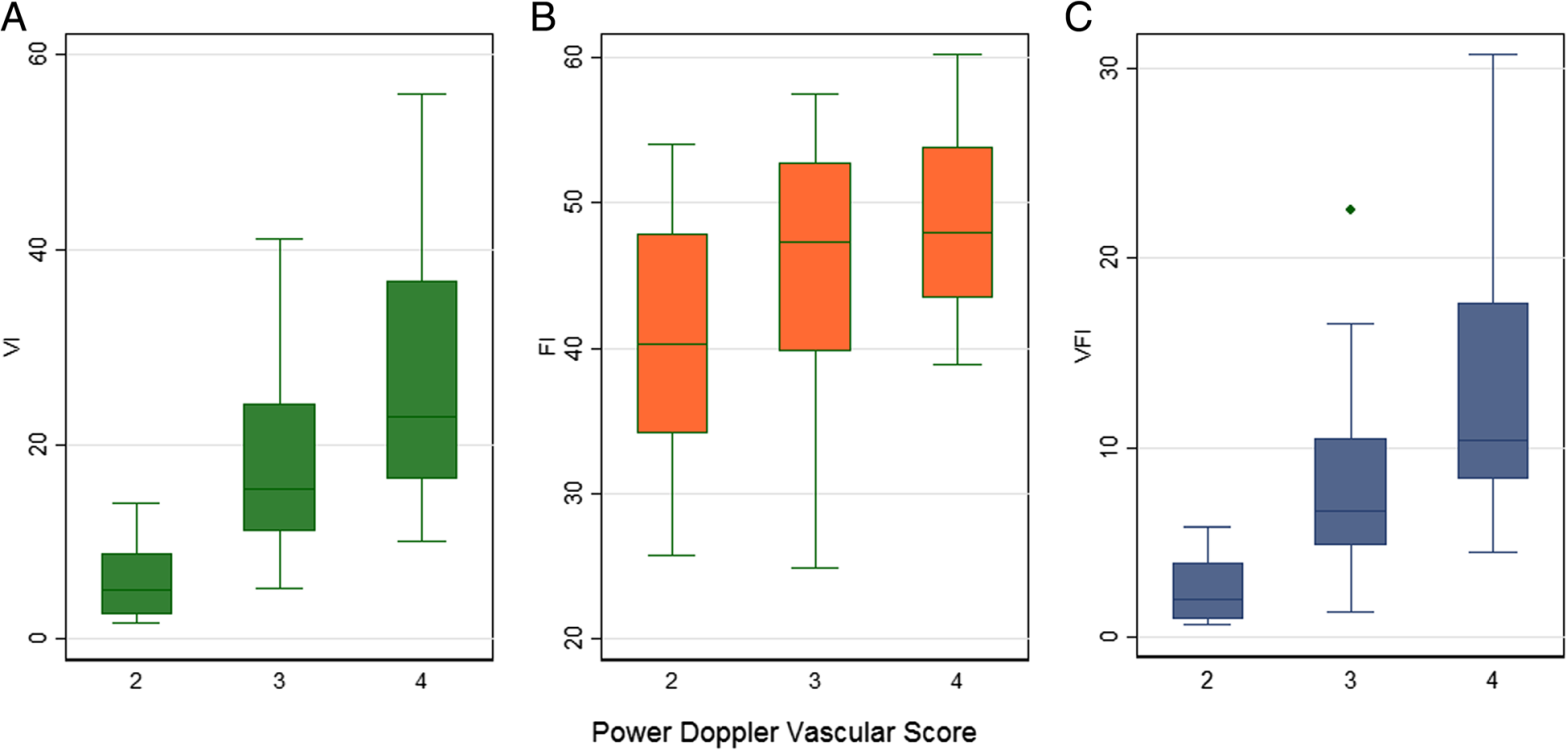
Distribution of 3D-PD indices according to power Doppler vascular scores. **a** vascularization index (VI), **b** flow index (FI), **c** vascularization-flow index (VFI)

Among the 49 cases with malignant or inconclusive results according to IOTA classification, 12 (24.5 %) presented vascular score two using conventional power Doppler, 21 (42.9 %) presented vascular score 3 and 16 (32.6 %) presented vascular score 4. The distribution of values for the 3D-PD indices according to conventional vascular scores is represented in Fig. 2. The median of VI was 5.0 for tumors with vascular score 2, 15.4 tumors with vascular score 3, and 22.8 for tumors with vascular score four (Kruskal-Wallis test, *P* = 0.0001). The median of FI was 40.3 for tumors with vascular score 2, 47.3 tumors with vascular score 3, and 48.0 for tumors with vascular score 4 (Kruskal-Wallis test, *P* = 0.06). The median of VFI was 2.0 for tumors with vascular score 2, 6.6 tumors with vascular score 3, and 10.4 tumors with vascular score 4 (Kruskal-Wallis test, *P* = 0.0001).

Based on the combination of limits of agreement and ROC curves, the sensitivity of 3D-PD presented considerable variation. Intra-observer variability can be reduced the sensitivity for VI from 100 % to 75 %, for FI from 100 % to 43 % for FI and from 100 % to 64 % for VFI.

## Discussion

Benign ovarian tumors can be safely managed by general gynecologists; however malignant ovarian tumors should be referred to a gynecologic oncology expert. The best management of “inconclusive” cases is still not defined; frequently the patients are referred to a gynecologic oncology expert and submitted to surgery. The major problem in the management of adnexal masses is to reduce the false positive rate without reducing the sensitivity. Our results show that 3D-PD angiography from the most complex area of adnexal mass can distinguish benign from malignant ovarian tumors. However, this method is not able to reduce the false positive rates of IOTA simple rules. The intra examiner repeatability is lower than the minimum recomended for secure clinical use and may impair significantly the accuracy of this method.

VI and VFI were more accurate to differentiate between benign and malignant ovarian tumors than FI. However, both indices were strongly associated with conventional power Doppler vascular scores. The variable power Doppler vascular score is used in the IOTA simple rules classification. Therefore, this association may be another reason why 3D-PD indices do not improve the accuracy of IOTA classification of ovarian tumors.

Despite the relatively small sample size, our study has the advantages of a planned prospective design and data collection by an experienced sonographer using a single machine with all set up parameters kept constant. Our observed values of 3D-PD indices were higher in malignant ovarian tumors which are in agreement with most of previously published data [23]. The effect of intra-observer variability of 3D-PD indices can be observed considering together the limits of agreement and the cut-offs from ROC curves. The sensitivity of the method can vary from 100 % to 43 % due to intra-observer variability, which in unacceptable for this group of patients. Some authors claim to have better reproducibility than we found in this series. Previous studies have shown intra-observer intraclass correlation coefficients (ICC) between 0.95-0.99 [9, 24, 25]. However, all these studies were based on single 3D-PD data-sets, which did not account for inherent clinical relevant sources of variability caused by respiratory and intestinal movements, and also from the phase of the cardiac cycle during acquisition [26].

The prevalence of malignant cases in our sample was high. In ultrasound settings linked to a gynecological oncology service the expected prevalence of malignancy in adnexal masses is higher than in general hospitals. Among our cases, 43 % were malignant, this prevalence is similar to the reported prevalence of 42 % of malignancy in oncology centers and 17 % in other centers [27].

Ovarian neoplasms are heterogeneous in morphology and even in genetic sub-clones of malignant cells [28]. Variable patterns of vascularization are frequently identified in different portions of the same tumor [29]. Therefore, diagnostic methods based on the analysis of parts of the tumor produce inevitable intra-observer variability. The key question is whether the disagreement is enough to cause confusion and impair the method validity.

## Conclusion

Our data provide strong evidence that 3D-PD angiography from spherical biopsy is not accurate for reducing the false positive rates of IOTA single rules.
